# Antibiotic Use in Surgical Wards: A Point Prevalence Survey Based on the WHO AWaRe Methodology

**DOI:** 10.3390/antibiotics15010012

**Published:** 2025-12-20

**Authors:** Jacopo Dolcini, Giorgia Maria Ricciotti, Giorgio Firmani, Lara Larcinese, Daniele Barbaresi, Ilaria Maria Faggi, Lucia Gatti, Anita Genga, Erlil Mali, Alex Marcello, Alessia Rinaldi, Oriana Dunia Toscano, Roberta Domizi, Marcello Mario D’Errico, Pamela Barbadoro

**Affiliations:** 1 Department of Biomedical Sciences and Public Health, Section of Hygiene, Preventive Medicine and Public Health, Polytechnic University of the Marche Region, 60126 Ancona, Italy; 2 Hospital Hygiene Unit, Azienda Ospedaliero-Universitaria delle Marche, 60126 Ancona, Italy; 3 Anesthesia and Intensive Care Unit, Department of Biomedical Sciences and Public Health, University of the Marche Region, via Tronto 10/a, 60126 Ancona, Italy

**Keywords:** surgical antibiotic prophylaxis, antimicrobial resistance, AWaRe classification, antibiotic stewardship, defined daily doses, cefazolin, Italy

## Abstract

**Background/Objectives**: In surgical antibiotic prophylaxis (SAP), most studies continue to report the number of prescriptions aggregated at the hospital level, rarely integrating the World Health Organization (WHO) Access, Watch, and Reserve (AWaRe) classes with standardized volume indicators. This study aimed to evaluate the utilization of antibiotics for SAP in a large Italian teaching hospital using both the number of prescriptions and defined daily doses (DDDs) and mapped the AWaRe models across different surgical specialties to highlight differences relevant to management. **Methods**: We conducted a prospective hospital-wide surveillance of all consecutive patients undergoing surgical procedures between March and May 2023 at the Azienda Ospedaliero-Universitaria delle Marche. Data included demographics, surgical specialty, and all antibiotic administrations with indication. For SAP, each prescription was classified according to the 2023 WHO AWaRe framework, and consumption was quantified using the WHO ATC/DDD methodology. **Results**: A total of 914 patients were monitored, with complete antibiotic data for 793 (86.8%). Among 433 SAP prescriptions, the most frequently used agent was cefazolin (82%), followed by amoxicillin/β-lactamase inhibitor (5%) and metronidazole (5%). According to AWaRe, 93% of SAP prescriptions were Access agents and 7% were Watch agents; no Reserve antibiotics were used. When expressed in DDDs (total: 443.5), 87.8% were Access and 12.2% Watch. Cefazolin accounted for over 85% of Access DDDs. **Conclusions**: By combining AWaRe classes with DDDs and resolving results by surgical specialty, this study extends hospital-level metrics and provides a pragmatic framework for SAP benchmarking. The predominance of Access agents is consistent with management objectives, while differences across specialties identify concrete tools for local quality improvement.

## 1. Introduction

Antimicrobial resistance (AMR) is one of the most urgent threats to global health, responsible for more than 1.2 million deaths annually and linked to widespread misuse of antibiotics [1]. Among hospital settings, surgical antibiotic prophylaxis (SAP) represents a major contributor to antibiotic consumption [2]. Indeed, surgical site infections (SSIs) represent one of the most frequent healthcare-associated infections (HAIs), accounting for up to 20% of all hospital-acquired infections and remaining a major cause of postoperative morbidity and mortality worldwide [3,4]. The occurrence of an SSI can significantly prolong hospitalization, increase the risk of reoperation, and raise healthcare costs, representing a key challenge for patient safety and resource management. In this context, timely and appropriate surgical antibiotic prophylaxis (SAP) is recognized as one of the most effective preventive measures, as emphasized by international guidelines [3,4,5]. Ensuring adherence to SSI prophylaxis guidelines is therefore essential for both patient outcomes and antimicrobial stewardship. Although its primary purpose is to prevent surgical site infections (SSIs), SAP is frequently prescribed inappropriately, with excessive duration, use of broad-spectrum agents, or non-adherence to guidelines [6,7,8]. These practices contribute to the development and spread of resistant organisms and decrease the effectiveness of first-line therapies [9]. To promote rational antibiotic use and guide stewardship initiatives, the World Health Organization (WHO) has developed the AWaRe classification system, which categorizes antibiotics into Access, Watch, and Reserve groups based on their spectrum, potential to induce resistance, and global relevance [10]. The WHO recommends that at least 60% of total antibiotic consumption should consist of Access antibiotics, which are typically narrow-spectrum and have a lower ecological impact [10,11]. The AWaRe framework has become an international reference for formulary design, procurement, and surveillance. Despite its relevance, the AWaRe classification is still underutilized in hospital-level evaluations of antibiotic use. In a recent systematic review [12], several indicators related to antibiotic utilization have been used in scientific literature; however, only a few are directly linked to the WHO AWaRe classification. Moreover, few studies have investigated antibiotic settings using such appropriate indicators. Previous studies on antibiotic consumption in hospital settings have primarily relied on aggregate prevalence indicators, often mixing therapeutic and prophylactic prescriptions, and rarely distinguishing the specific contribution of surgical prophylaxis. Moreover, most published works have reported proportions of AWaRe classes based on prescription counts only, without providing standardized volume metrics such as Defined Daily Doses (DDD). Few investigations have systematically assessed ward-wide data across all surgical specialties within a single institution, and SAP-focused AWaRe analyses remain underrepresented in the literature. In fact, most existing studies have used overall antibiotic consumption indicators without focusing on SAP [13,14], and AWaRe-based analyses are often limited to aggregate hospital-level data [14,15]. To address these gaps, the present study combines a prospective hospital-wide surveillance, a surgical prophylaxis–specific assessment, and a DDD-based stratification of antibiotic use by AWaRe class in the SAP context, thus providing a novel and more comprehensive benchmarking of stewardship indicators in the surgical setting.

Most hospital assessments of antibiotic use are currently based on hospital-level indicators and prescription numbers, often combining therapeutic and prophylactic exposures and rarely integrating WHO AWaRe classes with standardized volume indicators. SAP analyses performed by procedure or specialty remain underrepresented, limiting the feasibility of stewardship audits. The present study combined the AWaRe classification with DDD-based quantification to map SAP at both the hospital and specialty levels. Specifically, the study quantified SAP using the number of prescriptions and DDD, reported overall AWaRe distributions and distributions across surgical specialties, and highlighted where limited Watch exposure is focused. The approach of this study offers a pragmatic and replicable framework for assessing SAP, where decisions are made.

## 2. Results

A total of 914 patients (Table 1) undergoing surgical procedures were included in the surveillance, with data successfully collected for 793 cases (86.8%). The remaining 121 cases (13.2%) were classified as missing data. The completeness of data collection varied across departments, with some achieving nearly full coverage and others showing significant missing data. Among the surgical specialties, thoracic surgery achieved a 100% data collection rate, while plastic surgery had the highest percentage of missing data (34.4%). Other departments with a considerable proportion of missing cases included the neurosurgery division (22.9%) and urologic surgery (21.8%).

Table 2 presents demographic characteristics. The mean age of the patients was 62 years (SD = 17.67). Regarding gender distribution, 433 patients (55%) were male, while 360 patients (45%) were female. The average length of stay was 6.91 days (SD = 7.73).

**Figure 1:**
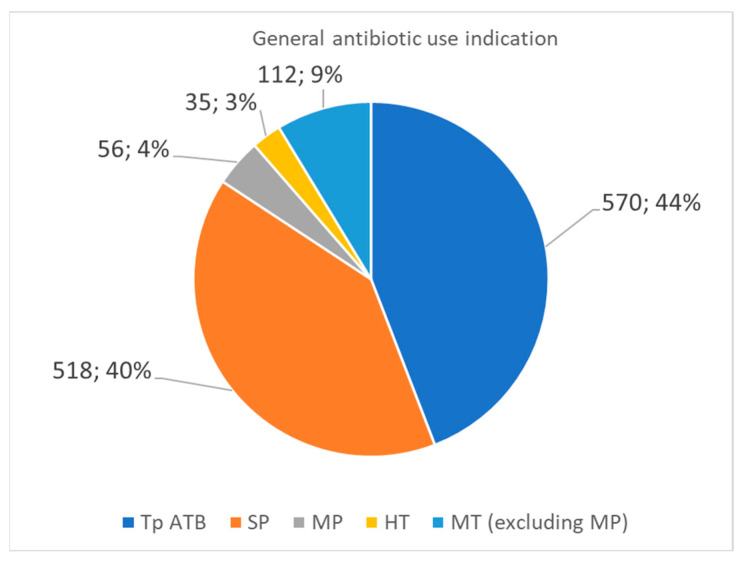
General antibiotic use indication: antibiotic therapy (Tp AtB), surgical prophylaxis (SP), medical prophylaxis (MP), home therapy (HT), medical therapy, excluding medical prophylaxis (MT excluding MP).

Figure 1 illustrates the distribution of antibiotic use among the surveyed patients. Antibiotic therapy (Tp ATB) was administered to 570 patients (44%), representing the most common category of antibiotic use. Surgical prophylaxis (PC) was applied in 518 cases (40%). A smaller proportion of patients (7%) received medical prophylaxis (PM), which refers to preventive antibiotic administration for non-surgical indications. Home antibiotic therapy (HT) was recorded in 3% of cases, suggesting that post-discharge antibiotic continuation was relatively rare. Lastly, medical therapy for non-prophylactic purposes (excluding PM) accounted for 9%, representing patients treated for therapeutic indications beyond surgical prophylaxis.

Focusing on surgical prophylaxis, a total of 433 antibiotic doses were administered. Among these prescriptions, the five most commonly used antibiotics were cefazolin (n = 354), amoxicillin with enzyme inhibitor (n = 23), metronidazole (n = 20), vancomycin (n = 8), and—tied in fifth place—ceftriaxone (n = 5), clarithromycin (n = 5), clindamycin (n = 5), and piperacillin with enzyme inhibitor (n = 5). The distribution of drugs used in surgical prophylaxis is detailed in Table 3.

According to the AWaRe classification, among the 433 prophylactic prescriptions, 404 (93.3%) belonged to the Access category, which includes narrow-spectrum antibiotics with a lower risk of resistance development. Within this group, cefazolin accounted for the vast majority (82% of the total prescriptions), followed by metronidazole, clindamycin, and amoxicillin with an enzyme inhibitor in smaller proportions. Only 29 prescriptions (6.7%) involved antibiotics classified as Watch, namely ceftriaxone, clarithromycin, piperacillin with enzyme inhibitor, vancomycin, teicoplanin, and cefuroxime. Importantly, no Reserve antibiotics—which are considered last-resort treatments—were used in this prophylactic context. This distribution is summarized in Table 4.

The distribution of prophylactic antibiotics across surgical specialties is reported in Table 5. Cefazolin was the most prescribed agent in almost all wards, consistently exceeding 80% of prophylactic prescriptions in cardiac, orthopedic, vascular, and thoracic surgery. Lower proportions were observed in maxillofacial, plastic, and urologic surgery, where broader-spectrum or additional agents such as metronidazole, clarithromycin, and third-generation cephalosporins were occasionally used. These findings indicate that the predominance of cefazolin observed at the hospital level was consistent across specialties, although with some variability in wards where clean-contaminated procedures were more common.

**Figure 2:**
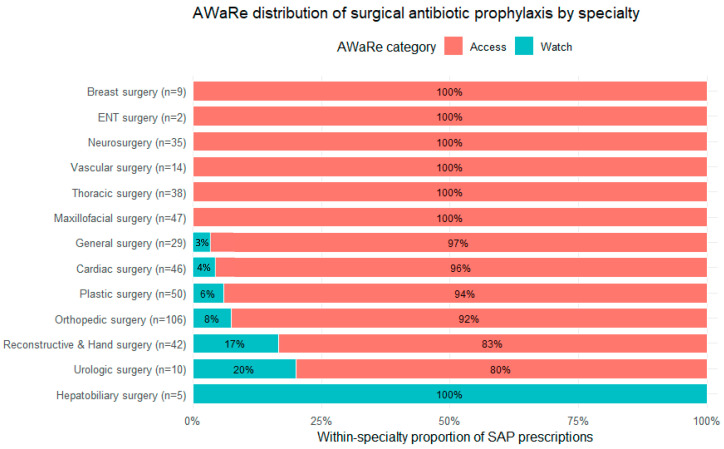
Specialty-level distribution of surgical antibiotic prophylaxis (SAP) prescriptions by AWaRe category. Bars represent within-specialty proportions (100% stacked) of Access and Watch antibiotics; specialty labels include the number of SAP prescriptions (n). Specialties are ordered by decreasing proportion of Watch antibiotics. Reserve antibiotics were not used.

A specialty-stratified distribution of AWaRe categories is shown in Figure 2 using a 100% stacked bar chart (within-specialty proportions). Overall, Access antibiotics predominated, while the specialty-level view highlights heterogeneity in Watch exposure: Hepatobiliary surgery showed 100% Watch (n = 5), followed by Urologic surgery (80% Access/20% Watch, n = 10) and Reconstructive & Hand surgery (83%/17%, n = 42). Smaller Watch proportions were observed in Orthopedic surgery (92%/8%, n = 106) and Plastic surgery (94%/6%, n = 50), while Cardiac surgery (96%/4%, n = 46) and General surgery (97%/3%, n = 29) displayed minimal Watch exposure. All remaining specialties had 100% Access prophylaxis.

### DDD-Based Quantification of Antibiotic Use

The most prescribed antibiotic by volume was cefazolin, with a total of 328.33 DDD, followed by amoxicillin with an enzyme inhibitor (55.0 DDD). Other notable contributions included oral clarithromycin (8.0 DDD), ceftriaxone (8.0 DDD), and piperacillin with an enzyme inhibitor (16.07 DDD). For many antibiotics, including meropenem (1.0 DDD), oral levofloxacin (1.0 DDD), and metronidazole (1.67 DDD), total consumption remained limited. A small number of agents, such as gentamicin (0.33 DDD) and ampicillin with an enzyme inhibitor (0.33 DDD), showed minimal usage. These figures reflect cumulative exposure across all patients receiving prophylaxis and serve as a baseline for assessing antibiotic volume and stewardship practices.

Table 6 shows the distribution of total antibiotic consumption for surgical prophylaxis expressed in Defined Daily Doses (DDD), categorized according to the WHO AWaRe classification. A total of 443.49 DDD were administered. Of these, 389.49 DDD (approximately 88%) were attributed to antibiotics in the Access group. The most frequently used Access antibiotic was cefazolin, accounting for 328.33 DDD. Other Access agents included amoxicillin with enzyme inhibitor (55.0 DDD), clindamycin (3.83 DDD), metronidazole (1.67 DDD), gentamicin (0.33 DDD), and ampicillin with enzyme inhibitor (0.33 DDD).

In contrast, Watch antibiotics accounted for a total of 54 DDD (12%). Among these, the most commonly used were piperacillin with enzyme inhibitor (16.07 DDD), teicoplanin (9.00 DDD), clarithromycin (8.50 DDD), and ceftriaxone (8.00 DDD). Other Watch antibiotics with lower DDD values included vancomycin (5.50 DDD), levofloxacin (3.00 DDD), ciprofloxacin (1.60 DDD), cefuroxime (1.33 DDD), and meropenem (1.00 DDD).

During the study period, the surgical cohort accumulated 5478 hospitalization days (bed-days). Based on this denominator, the overall antibiotic consumption for surgical prophylaxis corresponded to 8.10 DDD per 100 bed-days, indicating a low level of antibiotic use relative to total hospitalization time.

## 3. Discussion

This study provides a comprehensive overview of antibiotic use for surgical prophylaxis in a large university hospital in central Italy, with a focus on quantifying consumption through Defined Daily Doses (DDD) and evaluating compliance with the WHO AWaRe classification system [10,11]. The findings offer critical insights into local prescribing behaviors and their alignment with global antimicrobial stewardship targets.

The predominance of Access antibiotics in both the number of prescriptions (93%) and DDDs (88%) is particularly important. According to WHO guidelines, at least 60% of total antibiotic consumption should derive from Access antibiotics [10,11]. Our results not only exceed this threshold, but also demonstrate a strong reliance on cefazolin, which alone accounted for over 328 DDD, resulting in more than 85% of the Access antibiotic consumption. This is consistent with international surgical prophylaxis and SSI prevention guidelines, which recommend cefazolin as a first-line agent for many procedures [5].

Several contextual factors may further explain the high prevalence of cefazolin in our center. First, the demographic profile of our cohort, with a predominance of middle-aged and elderly patients, reflects surgical case-mix characteristics where cefazolin is strongly indicated. Second, several of the specialties included in our surveillance, such as orthopedic, vascular, and breast surgery, involve a high proportion of clean or clean-contaminated procedures for which international guidelines consistently recommend cefazolin as a first-line prophylactic agent [3,4,5]. Finally, the hospital has implemented dedicated stewardship measures in recent years, including formulary restrictions favoring Access antibiotics and periodic feedback to surgical teams, which likely contributed to the consistent adherence to guideline-recommended agents.

It is important, however, to interpret the WHO ≥ 60% Access target within the appropriate clinical context. This benchmark represents a population-level indicator encompassing all antibiotic consumption, whereas in surgical antibiotic prophylaxis (SAP), where guidelines mainly recommend Access agents, the expected proportion is naturally higher. Nevertheless, achieving 100% Access use is neither realistic nor necessarily desirable, as certain clinical circumstances (e.g., β-lactam allergy, MRSA colonization, or complex contaminated procedures) may justify the use of selected Watch antibiotics. The true stewardship objective in SAP should therefore focus on eliminating unjustified deviations rather than pursuing an absolute percentage of Access use.

The minimal use of Watch antibiotics, being 12% of total DDD, further supports the appropriateness of local prophylactic strategies. The most frequently used Watch agent was piperacillin with enzyme inhibitor (16.07 DDD), followed by teicoplanin (9.00 DDD) and clarithromycin (8.50 DDD). Although these drugs have a broader spectrum and a higher risk of selecting resistant pathogens, their relatively limited use is consistent with the stewardship goal of minimizing unnecessary exposure to high-risk agents. When standardized for hospitalization days, the total consumption corresponded to 8.10 DDD per 100 bed-days, a value consistent with WHO stewardship benchmarks and confirming the low intensity of antibiotic use in this surgical population. These findings compare favorably with those from previous international studies. For instance, data from several studies [14,16,17] reported that in many countries, Watch antibiotics represent 30–50% of total consumption in hospital settings, with third-generation cephalosporins, fluoroquinolones, and carbapenems being commonly overused. In this context, our 93% Access prescription rate places the present center well above the national average of 54.4% of Access antibiotic prescriptions detected in 2023 [18], suggesting effective local implementation of stewardship measures, especially when compared with the 65% target set by the Recommendation of the Council of the European Union [19].

Focusing more specifically on surgical prophylaxis, our findings reflect a more favorable AWaRe distribution than those observed in other practice-level reports, where broad-spectrum agents such as third-generation cephalosporins (e.g., ceftriaxone, cefuroxime) were among the most frequently inappropriately used antibiotics [7,8], sometimes exceeding the use of narrower-spectrum agents such as cefazolin in clinical practice. On the international level, a study by Sommerstein et al. in Swiss acute care hospitals re-ported that Watch antibiotics were prescribed in over 30% of prophylactic regimens, particularly cardiothoracic surgeries [20]. Compared to these data, our lower use of Watch agents and the high reliance on cefazolin suggest stronger adherence to stewardship-aligned practices and national recommendations [21].

However, some areas for potential optimization remain. The presence of 8.00 DDD of ceftriaxone, a third-generation cephalosporin categorized as Watch, raises concerns, especially if used in clean procedures or when narrower-spectrum alternatives are available. Similar considerations apply to the use of teicoplanin and vancomycin, whose routine use in prophylaxis should be carefully justified, particularly in non-MRSA-endemic surgical populations. These deviations from guideline-recommended prophylaxis may be explained by specific clinical contexts, such as patients with previous antibiotic exposure, beta-lactam allergies, known colonization with multidrug-resistant organisms (e.g., MRSA), or high-risk procedures where surgeons may opt for broader-spectrum agents as a precautionary measure. Additionally, differences in surgical team practices and the lack of real-time stewardship feedback during the perioperative phase may contribute to such variations. Although such deviations were rare, they may contribute to selective pressure for resistance and highlight the importance of continuous antimicrobial stewardship efforts. Continuous education and feedback interventions are being reinforced to further standardize SAP practices and minimize unnecessary use of Watch antibiotics.

Finally, the use of DDD as a quantification tool complements the analysis of raw prescription counts and allows for a more standardized comparison with international benchmarks. It is important to acknowledge, however, that DDDs may overestimate exposure for some antibiotics with lower recommended prophylactic doses, and they do not capture appropriateness at the individual patient level.

Among the strengths of this study is the use of standardized indicators based on the WHO AWaRe classification, as recently recommended by Funiciello et al. (2024) in their systematic review [12]. These indicators allow for robust benchmarking of antibiotic use and are particularly well-suited for institutional audits and stewardship interventions. Another strength is the detailed, manual validation of DDD calculations based on real-world prescribing data, ensuring accuracy and minimizing classification errors.

The study also benefits from its comprehensive inclusion of all surgical units within a large tertiary hospital, offering a wide view of real-world clinical practices across multiple specialties.

However, several limitations should be considered, particularly for researchers aiming to replicate this type of surveillance. Firstly, the study was conducted in a single tertiary hospital over a limited observation window; therefore, the findings may not fully capture seasonal variation, temporal trends, or prescribing changes over time. Secondly, the surveillance dataset did not include procedure-level quality indicators that are central to SAP appropriateness, such as timing of administration relative to incision, weight-based dosing, intraoperative re-dosing in prolonged procedures, and discontinuation at wound closure, so adherence to guideline-recommended processes could not be formally assessed. Future PPS-based assessments should therefore be complemented by targeted audits capturing these dimensions, as highlighted in recent multidisciplinary recommendations on optimizing SAP [8]. Thirdly, patient-level clinical data were limited; consequently, we could not analytically distinguish appropriate from inappropriate use of Watch agents, because key justifications (e.g., documented β-lactam allergy, MRSA colonization status, degree of contamination, prior antibiotic exposure) were not available. Fourthly, although DDDs enable standardized quantification and benchmarking [22], they remain an aggregate indicator and may not perfectly reflect prophylactic dosing regimens at the individual level; thus, DDD-based results should be interpreted as consumption metrics rather than direct measures of appropriateness. Fifthly, data completeness varied across specialties, reflecting differences in documentation and case volume, which may have influenced ward-level estimates. Finally, microbiology data and postoperative outcomes (e.g., SSI incidence, other HAIs, ICU admission, mortality) were not linked to antibiotic use in this analysis; future longitudinal studies integrating utilization metrics with outcome surveillance would provide stronger evidence on the clinical impact of SAP patterns. These clinical outcomes may represent an important upgrade for future longitudinal studies integrating antibiotic utilization data with infection surveillance indicators.

In conclusion, this hospital’s surgical prophylaxis practices align closely with WHO recommendations in terms of AWaRe distribution, showing a high proportion of Access antibiotic use and limited reliance on Watch agents. By quantifying both adherence and deviations from recommended regimens, this study provides actionable evidence to guide ongoing stewardship initiatives and strengthen SSI prevention strategies within the surgical department. These findings reinforce the importance of local stewardship programs and highlight the value of AWaRe-based metrics in monitoring and guiding antibiotic use in surgical settings.

### Practical Implications for Antimicrobial Stewardship

This study adds practical value to the evaluation of surgical antibiotic prophylaxis (SAP) by providing a reproducible stewardship-oriented reporting framework. First, it combines the WHO AWaRe classification with ATC/DDD-based metrics specifically for SAP, enabling standardized quantification of prophylactic exposure beyond simple prescription counts. Second, it reports AWaRe patterns at the level of surgical specialty, which helps identify clinically relevant heterogeneity that can be obscured by hospital-wide aggregates (e.g., an overall predominance of Access antibiotics alongside concentrated use of Watch agents in selected specialties). Third, this specialty-level “counts + DDD + AWaRe” approach can be readily replicated in routine audit cycles to support targeted feedback, prioritization of improvement actions, and benchmarking over time and across comparable surgical settings, even in healthcare systems with different organizational structures.

## 4. Materials and Methods

### 4.1. Study Design, Setting, and Study Population

We conducted a prospective, hospital-wide surveillance of antibiotic use embedded in the routine healthcare-associated infection (HAI) prevention and antimicrobial stewardship program at the Azienda Ospedaliero-Universitaria delle Marche (Marche Region, Italy). All consecutive patients undergoing a surgical procedure between March and May 2023 in the following surgical specialties/wards were eligible: Cardiac Surgery, Hepatobiliary Surgery, Maxillofacial Surgery, Plastic Surgery, Reconstructive and Hand Surgery, Thoracic Surgery, Vascular Surgery, Neurosurgery, Orthopedic Surgery, Urologic Surgery, General Surgery, Otorhinolaryngology, and Breast Surgery. Patients were included if they underwent surgery during the surveillance period and had an available ward-level record allowing linkage of antibiotic administrations to an indication. For the present analysis, we focused on antibiotics administered with the indication of surgical prophylaxis (SAP). Antibiotics administered for therapeutic indications (e.g., treatment of postoperative infections such as SSI, pneumonia, urinary tract infection) were excluded from SAP analyses.

### 4.2. Data Sources and Data Collection Procedures

Data were collected at the patient level from routine clinical documentation and medication administration records, using a standardized extraction form developed for the surveillance program. Collected variables included age (years), sex, admission and discharge dates, surgical specialty/ward, procedure type (as recorded in the clinical record), and antibiotic administrations. Indications were categorized as therapeutic antibiotic therapy, surgical prophylaxis, medical prophylaxis, home therapy (post-discharge continuation), and medical therapy excluding medical prophylaxis.

To improve standardization, data collectors followed written instructions defining indication categories and rules for attribution. Ambiguous records were reviewed and resolved through discussion within the surveillance team, prioritizing the indication documented in the medical chart and/or perioperative orders. When the indication could not be reliably classified, the record was retained in the overall denominators but excluded from SAP-specific analyses. The activity was conducted within the routine HAI surveillance and stewardship program. Data were analyzed in a de-identified, aggregated form with no patient-level identifiers. Formal Ethics Committee review was waived according to local policy; the study was conducted in accordance with the Declaration of Helsinki and GDPR principles.

### 4.3. Handling of Missing, Ambiguous, and Inconsistent Information

Missing data were not imputed. We report ward-specific completeness and calculate percentages using non-missing denominators for each analysis. Inconsistencies (e.g., missing dose information for a documented prophylactic administration) were handled as follows: administrations without sufficient information to compute grams were included in prescription counts but excluded from DDD computation for that molecule. “Home therapy” (HT) was defined as antibiotic continuation after discharge/outpatient prescription and was not considered part of perioperative SAP; therefore, HT was reported descriptively in the overall indication distribution but excluded from all SAP-specific AWaRe/DDD analyses.

Patient-level clinical variables (e.g., ASA class, comorbidities, smoking status, allergy history, colonization with resistant organisms) were not systematically available in the routine dataset and were therefore not included.

### 4.4. AWaRe Classification

Each administered antibiotic was mapped to the WHO AWaRe 2023 categories (Access, Watch, Reserve) based on molecule and route of administration. For SAP, we reported: the number and percentage of administrations by AWaRe category and AWaRe stratification of total consumption expressed in DDDs.

### 4.5. Statistical Analysis

Antibiotic use was summarized overall and by surgical specialty as counts (N) and percentages. For SAP, we reported molecule-level distributions, AWaRe category distributions, and DDD-based consumption metrics (total DDD and DDD/100 bed-days). All analyses used non-missing denominators.

### 4.6. Defined Daily Dose (DDD) Methodology

Antibiotic consumption for surgical prophylaxis was standardized using the WHO ATC/DDD methodology [22]. For every molecule and route of administration actually used (parenteral or oral), we retrieved the corresponding ATC code and WHO DDD value (grams per day) for that route. Fixed-dose combinations (e.g., amoxicillin/clavulanate; ampicillin/sulbactam) were handled using the combination-specific ATC code and its WHO DDD. For each drug, the number of DDDs was calculated as

(1) $$DDD=\frac{total\;grams\;administered\;across\;all\;prophylaxis\;doses}{WHO\;DDD\;(grams)\;for\;that\;molecule\;and\;route}$$

Total grams were obtained from the administered dose (in grams) multiplied by the number of doses/vials recorded during the observation period. DDDs were then summed by molecule and aggregated by AWaRe class to obtain total DDDs per molecule and total DDDs per AWaRe class for surgical prophylaxis. In addition, to align with WHO reference metrics, total antibiotic consumption was expressed as DDD per 100 bed-days, calculated as the ratio between the total DDDs administered and the total hospitalization days (bed-days) of all surgical patients included in the surveillance, multiplied by 100.

## 5. Conclusions

This point prevalence survey highlights a favorable pattern of surgical antibiotic prophylaxis at a large Italian teaching hospital, with a predominance of Access antibiotics and minimal use of those included in the Watch class. Both prescription-based and DDD-based indicators exceeded WHO stewardship targets, reflecting strong adherence to national and international recommendations and confirming the central role of cefazolin in local protocols. Occasional deviations involving Watch antibiotics such as ceftriaxone, teicoplanin, or vancomycin likely reflect specific clinical circumstances (e.g., allergy, MRSA risk, or complex procedures) rather than systematic noncompliance. Nevertheless, such deviations may still contribute to an increased risk of inappropriate prescriptions and the development of multidrug-resistant bacteria. Continuous stewardship activities—including clinician education, audit-feedback mechanisms, and protocol harmonization—remain essential to sustain appropriate prophylaxis and minimize the emergence of antimicrobial resistance. The present findings reinforce the value of AWaRe-based indicators as practical tools for monitoring adherence and guiding targeted stewardship interventions in surgical settings.

## Figures and Tables

**Table 1:** Sample size and wards.

Department	Sample Size	Collected	Missing	Collected (%)	Missing (%)
Breast Surgery	60	53	7	88.3	11.7
Cardiac Surgery	72	64	8	88.9	11.1
General & Hepatobiliary Surgery	131	111	20	84.7	15.3
Head & Neck Surgery	73	67	6	91.8	8.2
Neurosurgery	118	100	18	84.7	15.3
Orthopedic Surgery	126	119	7	94.4	5.6
Plastic & Reconstructive Surgery	131	99	32	75.6	24.4
Thoracic Surgery	49	49	0	100.0	0.0
Urologic Surgery	78	61	17	78.2	21.8
Vascular Surgery	76	70	6	92.1	7.9
Total	914	793	121	86.8	13.2

**Table 2:** Patients’ characteristics.

Variable	Mean	SD
**Age**	62	17.67
**Length of stay**	6.91	7.73
**Gender**	**N**	**%**
Male	433	55
Female	360	45

**Table 3:** Distribution of the most common antibiotics used in surgical prophylaxis.

Antibiotic	N	%
Cefazolin	354	81.8
Amoxicillin with enzyme inhibitor	23	5.3
Metronidazole	20	4.6
Vancomycin	8	1.8
Ceftriaxone	5	1.2
Clarithromycin	5	1.2
Clindamycin	5	1.2
Piperacillin with enzyme inhibitor	5	1.2
Teicoplanin	2	0.5
Cefixime	2	0.5
Gentamicin	1	0.2
Levofloxacin	1	0.2
Amoxicillin	1	0.2
Cefuroxime	1	0.2

**Table 4:** Distribution of antibiotics by AWaRe category.

AWaRe Category	N	%	Molecules
Access	404	93.3	Amoxicillin with enzyme inhibitor, Cefazolin, Clindamycin, Metronidazole
Watch	29	6.7	Ceftriaxone, Clarithromycin, Piperacillin with enzyme inhibitor, Vancomycin, Teicoplanin, Cefixime, Cefuroxime

**Table 5:** Distribution of prophylactic antibiotics by surgical specialty.

Specialty/Ward	Total SAP Prescriptions (n)	Cefazolin n (%)	Amoxicillin/β-Lactamase inh. n (%)	Metronidazole n (%)	Other Access n (%)	Watch Antibiotics n (%)
Cardiac surgery	46	44 (95.7)	-	-	-	2 (4.3)
Hepatobiliary surgery	5	0 (0.0)	-	-	-	5 (100.0)
Maxillofacial surgery	47	31 (66.0)	2 (4.3)	14 (29.8)	-	-
Plastic surgery	50	42 (84.0)	5 (10.0)	-	-	3 (6.0)
Reconstructive & hand surgery	42	35 (83.3)	-	-	-	7 (16.7)
Thoracic surgery	38	33 (86.8)	-	-	5 (13.2)	-
Vascular surgery	14	12 (85.7)	2 (14.3)	-	-	-
Neurosurgery	35	34 (97.1)	1 (2.9)	-	-	-
Orthopedic surgery	106	97 (91.5)	1 (0.9)	-	-	8 (7.6)
Urologic surgery	10	6 (60.0)	-	-	2 (20.0)	2 (20.0)
General surgery	29	20 (69.0)	2 (6.9)	6 (20.7)	-	1 (3.4)
ENT surgery	2	-	1 (50.0)	-	1 (50.0)	-
Breast surgery	9	-	9 (100.0)	-	-	-
Total	433	354 (81.7)	28 (6.5)	20 (4.6)	8 (1.9)	29 (6.7)

Note: Watch antibiotics include ceftriaxone, clarithromycin, piperacillin–tazobactam, vancomycin, teicoplanin, levofloxacin, ciprofloxacin, and cefixime.

**Table 6:** Antibiotics DDD stratified by Access (green), Watch (yellow), and Reserve Class.

Class	Antibiotic	DDD
Access	Amoxicillin and an enzyme inhibitor	55.0
	Ampicillin and enzyme inhibitor	0.33
	Cefazolin	328.33
	Clindamicin	3.83
	Gentamicin	0.33
	Metronidazole	1.67
	Totale Access	389.49
Watch	Ceftriaxone	8.00
	Cefuroxime	1.33
	Ciprofloxacin	1.60
	Claritromicin	8.50
	Levofloxacin	3.00
	Meropenem	1.00
	Piperacillin and enzymatic inhibitor	16.07
	Teicoplanin	9.00
	Vancomycin	5.50
	Total Watch	54

## Data Availability

The raw data supporting the conclusions of this article will be made available by the authors on request.
